# Immunotherapy and Asthma in Children

**DOI:** 10.3389/fped.2018.00231

**Published:** 2018-08-21

**Authors:** Maria A. Tosca, Amelia Licari, Roberta Olcese, Gianluigi Marseglia, Oliviero Sacco, Giorgio Ciprandi

**Affiliations:** ^1^Department of Pediatrics, Allergy Center, Istituto Giannina Gaslini (IRCCS), Genoa, Italy; ^2^Department of Pediatrics, Ospedale San Matteo (IRCCS), Pediatrics Clinic, University of Pavia, Pavia, Italy; ^3^Pediatric Pulmonology and Endoscopy, Istituto Giannina Gaslini (IRCCS), Genoa, Italy; ^4^Allergy Clinic, Ospedale San Martino (IRCCS), Genoa, Italy

**Keywords:** allergen immunotherapy, subcutaneous immunotherapy, sublingual immunotherapy, allergic rhinitis, asthma

## Abstract

Allergen immunotherapy (AIT) is still the only disease-modifying treatment strategy for IgE-mediated allergic diseases, with consolidated evidence both in adults and children. AIT is effective in determining clinical improvement of allergic rhinitis and asthma, such as reduced symptoms, medication use, and improvement of quality of life, with a long-lasting effect after cessation of treatment. Results from recent clinical studies have implemented the evidence of effectiveness and safety of allergen immunotherapy for the treatment of allergic asthma, so that the current asthma guidelines now recommend sublingual immunotherapy as an add-on therapy for asthma in adults and adolescents with house dust mite allergy, allergic rhinitis, and exacerbations despite low-to-moderate dose ICS, with forced expiratory volume in 1 second more than 70% predicted. AIT may also reduce the risk of progression from allergic rhinitis to asthma in children and prevent the onset of new sensitizations, thus representing a potentially preventive method of treatment. The aim of this review is to present an updated overview of the clinical indications of AIT, with particular reference to pediatric asthma, of the mechanisms of clinical and immunological tolerance to allergens, and of the potential biomarkers predicting clinical response.

## Introduction

Allergen immunotherapy (AIT) is the administration of the causal allergen to control allergic inflammation and symptoms. AIT has been used for over a century and it is actually considered the only disease-modifying treatment strategy for IgE-mediated allergic diseases, as causing a persistent immunological and clinical tolerance toward the causal allergen (1).

Both subcutaneous AIT (SCIT) and sublingual (SLIT) are used and accepted as effective treatments for adults and children with allergic rhinitis (AR) with or without asthma (2, 3). Historically, SCIT was early proposed as the first route of AIT administration, since first report (4). However, despite its proven effectiveness, the use of SCIT is still limited by the need for frequent injections by a doctor over a minimum of 3 years, and, mostly important, the potential occurrence of systemic severe reactions. Consequently, SCIT should be administered in a medical setting by clinicians able to manage anaphylaxis (1). Furthermore, the risk of systemic reactions to SCIT is greater in subjects with uncontrolled asthma and with accelerated dosing schedules (5). Given these disadvantages, SLIT may represent a viable alternative to SCIT, mainly in children, allowing safe self-administration at home (6–8). So, route selection vaccine is based on availability or approval, cost, and the patient's age or the physician's or patient's preference (9).

SCIT or SLIT products cannot be actually compared due to their heterogeneous composition and allergen concentration (10). Existing studies suggest that both may induce similar immunologic changes (11). Allergens are used for SCIT as aqueous or physically-adsorbed (depot) extracts, as well as chemically modified allergens (allergoids) as depot formulations. Allergens for SLIT are used as drops or tablets. Waiting for a harmonized and international AIT products regulation, they are still available most commonly either by being distributed as “named patient products” (NPP), prepared simply in compliance with Good Manufacturing Practice, and by obtaining a formal marketing authorization (12). SLIT tablets for grass pollen and house dust mite (HDM) have been recently registered for use in children, adolescents, and adults (13, 14).

Through complex molecular and cellular mechanisms inducing immune tolerance, effective AIT may modify the natural course of allergic disease, both preventing the onset of new sensitizations and the clinical disease progression (from rhinitis to asthma). AIT may also control allergic symptoms that are unresponsive to avoidance strategies and medications, reduce medication use, and improve quality of life, with a long-lasting effect after cessation (15, 16). Results from recent trials have implemented the evidence of effectiveness and safety of AIT for the treatment of allergic asthma, so that updated asthma guidelines now recommend SLIT as an add-on therapy for asthma in adults and adolescents with HDM allergy, under certain conditions (3).

The aim of this review is to present an updated overview of the clinical indications of AIT, with particular reference to pediatric asthma, of the mechanisms of clinical and immunological tolerance to allergens, and of the potential biomarkers predicting clinical response. A literature search was performed through Medline via Pubmed to identify all relevant articles published in English, on the basis of the following three search terms: “allergen immunotherapy”, “children,” and “asthma.” From the articles retrieved in the first round of search, additional references were identified by a manual search among the cited references.

## Overview of the mechanisms of allergen immunotherapy

AIT targets the upper and lower respiratory allergic symptoms by modulating the IgE-mediated response consequent to allergen exposure. Through multiple mechanisms involving both innate and adaptive immunity, AIT regulates T- and B-cells, changing antibody isotypes, decreases mediator release, and migration of inflammatory cells to tissues (17–19).

A key mechanism in inducing immunologic tolerance is the upregulation of allergen-specific T-regulatory (Treg) cells and B-regulatory (Breg) cells, which primarily down-regulate the Th_2_ response (19) (Table 1). Regulatory cells inhibit the activation of allergen-specific Th_2_ lymphocytes, suppress allergic inflammation, and ultimately shift toward a Type 1-mediated immune response, releasing cytokines, interleukin (IL)-10 and transforming growth factor-β (TGF-β) (20).

**Table 1:** Mechanisms of immunologic tolerance mediated by T and B regulatory cells during allergen immunotherapy.

**Treg-mediated mechanisms**
Release regulatory cytokines (IL-10, TGF-β, and IL-35)
Induce tolerogenic DCs subsets
Reduced number of ILC2
Suppress activation of allergen-specific Th_2_ lymphocytes
Downregulate the expression of FCεRI receptors on mast cells
Decrease allergen-specific IgE synthesis
Promote B-cell production of IgG_4_ antibody
**Breg-mediated mechanisms**
Release regulatory cytokines (IL-10, TGF-β)
Induce the synthesis of IgG4 blocking antibodies
Inhibit activation and proliferation of effector T lymphocytes
Suppress Th_2_-dependent inflammation
Promote T-cell expression of Foxp3 and generation of functional Treg cells

*Breg, B regulatory; DCs, dendritic cells; FCεRI, high-affinity receptor for the Fc region of IgE; Foxp3, forkhead box P3; IgE, immunoglobulin E; IgG_4_, immunoglobulin G subtype 4; IL, interleukin; ILC2, innate lymphoid cells type 2; TGF, transforming growth factor; Th_2_, T helper type 2; Treg, T regulatory*.

After high-dose allergen administration by AIT, dendritic cells (DCs) produce IL-12, IL-27, and IL-10, generating and activating distinct phenotypes of Tregs: natural (nTreg) and inducible (iTreg) cells (17, 19). Both nTreg and iTreg cells suppress allergic response through direct and indirect mechanisms: release regulatory cytokines (IL-10, TGF-β, and IL-35), directly induce tolerogenic DCs subsets, suppress activation of allergen-specific Th_2_ lymphocytes, downregulate the expression of FCεRI receptors on mast cells, decrease allergen-specific IgE synthesis, and promote B-cell production of IgG4 in an allergen-independent manner (17, 19). IL-10 directly inhibits T-cell-associated cytokines, including IL-4 and IL-5, reduces proinflammatory cytokine from mast cells and eosinophils, decreases allergen-specific IgE production, and increases IgA and IgG_4_ levels (19). Competitively binding to the same site epitopes recognized by IgE, IgG_4_ exert a sort of “immunologic blockade” inhibiting mast cell and basophil degranulation. In addition, IgG_4_ has been proposed to co-stimulate the inhibitory IgG receptor FcγRIIb, which can negatively regulate FcεRI signaling and in turn inhibit effector cell activation. IgG_4_ also inhibit IgE-mediated facilitation of allergen presentation to T lymphocytes (20). TGF-β suppresses both activity and proliferation of Th_2_ cells and innate lymphoid cells type 2 (ILC2), thus inhibiting Th_2_-cytokines (IL-4, IL-5, IL-9, and IL-13), and consequently decreasing the activation of eosinophils, basophils, mast cells and IgE-secreting B lymphocytes (17, 19).

Bregs play a key role in inducing immune tolerance to allergens, directly promoting the synthesis of allergen-specific IgG_4_, inhibiting activation and proliferation of effector T lymphocytes, suppressing Th_2_-dependent inflammation, increasing Treg cells (21). Overall, AIT is able to restore the impaired allergen tolerance.

## Clinical indications of AIT in children with respiratory allergy

AIT should be considered in patients who have AR with or without conjunctivitis, and/or asthma with documented sensitization consisting with symptoms after sensitizing-allergen exposure (22). Candidates for AIT are patients with uncontrolled symptoms by medications and/or environmental prevention or experiencing drug adverse effects or wishing reduction of long-term treatment (22).

The evidence of efficacy and safety of AIT in AR and/or asthma have been documented both in adults and children (6, 23, 24).

### Allergic rhinitis

AIT is the established treatment of choice for AR patients experiencing failed allergen avoidance and/or medical therapy (2, 25). European Academy of Allergy and Clinical Immunology (EAACI) 2018 guidelines recommend AIT in AR patients, with or without conjunctivitis, with evident sensitization to one or more clinically relevant allergens and moderate-to-severe symptoms despite regular and/or avoidance strategies (6, 26). This statement is based on a body of international trials evidence providing a comprehensive assessment of AIT in AR: both SCIT and SLIT are effective for seasonal and perennial AR in achieving short-term improvements in symptom, medication use, and combined symptom and medication scores (27, 28). The evidence of longer-term effectiveness is documented for grass AIT, especially for tablets (26). Thus, standardized and validated AIT products should be used when available, and a product-specific evaluation of evidence is actually recommended before initiating treatment with a specific product (6, 26).

With particular reference to children, AIT should be considered similarly to adults (26). Although major gaps still exist for the pediatric age (29), it can be actually recommended: (i) continuous, pre- and pre/co-seasonal SCIT for children with seasonal AR; (ii) continuous SCIT for perennial AR (weak recommendation due to the lack of exclusive pediatric data); (iii) pre-co-seasonal and continuous SLIT for seasonal AR (both tablet and drop); (iv) poor evidence for perennial AR, although the effectiveness of SLIT tablet approach has been demonstrated in the short term in mixed adult/adolescent studies (26). It is also recommended a minimum of 3-year course to achieve long-term efficacy (6).

### Asthma

Asthma is a common chronic disease affecting all age groups, with up to 20% of children aged 6–7 years experiencing severe wheezing episodes within a year (30). The actual asthma management is control-based: therapeutic strategies are based on a stepwise approach and adjusted in a continuous cycle involving assessment, treatment and review (3, 31–33). However, standard pharmacotherapy does not affect the underlying pathogenetic immune response, as it is withdrawn symptoms and inflammation occur again.

SLIT is actually recommended as add-on treatment option in adult HDM-sensitized asthmatics with concomitant AR who have exacerbations despite inhaled corticosteroids (ICS] treatment, with forced expiratory volume in 1 s (FEV_1_) more than 70% predicted, as stated in the latest Global Initiative for Asthma Report (GINA) update (3). Asthma must be mild-to-moderate and allergic, and well or “partially” controlled by standard pharmacotherapy; asthma control has to be maintained throughout AIT course (34). Conversely, AIT is still restricted in patients with uncontrolled asthma, as it represents a significant risk factor for serious and even fatal adverse reactions (5). Coupling anti-IgE biological therapy (omalizumab) with AIT has been proposed as a suitable option to increase SCIT effectiveness and safety (35–38).

This significant change in the GINA strategy draws upon recent Phase III clinical trial evaluating the treatment of asthma with the standardized quality (SQ) HDM SLIT-tablet in adults: the addition of HDM SLIT to maintenance therapies reduced ICS or the time to first exacerbation upon ICS reduction, suggesting that SQ HDM SLIT-tablet may improve overall asthma control (39). While these data require further studies to confirm long-term efficacy and safety of this product in adults, less information is available for adolescents (40, 41) and studies are in progress in children (42). HDM sensitization in early childhood represents an important risk factor for the development of asthma (43) and is linked to the impairment of lung function in school-age asthmatic children (43, 44) and asthma persistence into adulthood (45). So, other single HDM SLIT preparations have been tested in children and adolescents with allergic asthma. Among others, SLIT with the 300 index of reactivity (IR)-standardized HDM extract, in children aged 6–18 years with AR with or without allergic asthma, improved rhinitis and/or asthma symptoms scores, together with a reduction of rescue medications (46).

Meta-analyses confirmed the effectiveness and the safety profile of AIT in allergic asthma for adults and children (27, 47–51), mainly concerning SLIT in children (22, 52–54). Consistent reduction in combined AR and asthma symptom and medication scores have been demonstrated in pediatric patients with asthma and comorbid AR treated with SLIT (48, 53, 55). These clinical effects have been also demonstrated to be persistent after AIT discontinuation up to 5 years (56). SCIT has demonstrated effectiveness in controlling asthma and reducing medication use (49, 50, 57). Meta-analyses of randomized clinical trials using SCIT in asthma have demonstrated a significant reduction in symptoms, medication use, and AHR both in children and adults, while the effect on lung function showed conflicting results (49, 50). Studies conducted in children showed similar results (58–61). SCIT may have a long-term impact on childhood asthma, as demonstrated in a prospective study using HDM SCIT: after 3 years of SCIT discontinuation, a global remission of asthma (in particular reduced doses of ICS, lower asthma symptom scores, higher quality of life scores, less AHR, and higher FEV_1_) was reported in treated patients compared to controls (62). In another retrospective study, asthmatics allergic to either HDM or grass pollen, treated with SCIT in childhood, were re-evaluated 9 years after the discontinuation, showing a three-time lower risk of frequent asthma symptoms than controls, but without any difference in lung function or medication use (63).

Overall, both SCIT and SLIT appear to be effective for the treatment of AR and asthma in children (64, 65). Of particular interest for the pediatric population, it is the AIT ability (both SCIT and SLIT) to reduce ICS doses together with the impact on asthma control. These promising results highlight the immunomodulating pivotal role of AIT in controlling and inducing remission of disease activity. Furthermore, the persistence of these clinical effects after discontinuation separates AIT uniquely from other anti-allergic therapies.

### Prevention of allergy progression and asthma onset

Over the last decade, the disease-modifying properties of AIT have been largely investigated, mainly focusing on the prevention of allergic sensitization and asthma onset.

Developing new sensitizations is characteristic of the natural history of allergy. A preventive effect of AIT on the onset of new sensitizations has been reported (1, 66) and demonstrated in asthmatic children mono-sensitized to HDM (67, 68). However, a recent systematic review and meta-analysis on this topic reported a low level of evidence related to the heterogeneity and the high risk of bias of the included studies (69, 70). A recent EAACI-funded meta-analysis highlighted a reduced risk of developing new sensitizations at least over the short period, but none on the long-term (71).

Children with AR have an increased risk of developing asthma later on in life when compared to those without AR, especially those with AHR (72). Few but significant studies investigated disease modification in children, mainly concerning AIT (73–77). The prevention of allergy (PAT) study was a large prospective randomized controlled study to evaluate the preventive effect of SCIT in children (aged 6–14 years) with grass and/or birch AR without asthma (73–75). Actively-treated children had a significantly reduced risk of developing asthma and fewer asthma symptoms after 3-year treatment compared to controls (73). This preventive effect persisted at 5 years (74) and 7 years (75) after SCIT discontinuation. The preventive effect of SLIT was evaluated in two open trials conducted on children with AR with or without asthma: a 3-year course of SLIT improved AR symptoms, reduced onset of asthma, and decreased AHR (78, 79). Although promising, these findings had a major limitation as derived from open studies with a limited number of subjects. The results of the grass tablet asthma prevention (GAP) study, the first randomized, double-blind, placebo-controlled trial, have been recently published (77). The GAP study involved 812 children (aged 5–12 years), with grass-pollen AR and without asthma, who received 3-year SQ grass-SLIT-tablet or placebo and were followed for 2 years after discontinuation (77). Treated children significantly reduced the risk of experiencing asthma symptoms or using asthma medication, as well as AR-related symptoms and medications, at the end of the trial, during the 2-year post-treatment follow-up, and during the entire 5-year trial period (77). Taken together, these studies suggest that AIT might reduce the risk of developing asthma symptoms in children, especially in those with AR. EAACI guidelines on the PAT recommend a 3-year course of AIT in children with moderate-to-severe AR and grass/birch pollen allergy, uncontrolled with pharmacotherapy for short-term and possibly long-term prevention of asthma symptoms in addition to improving the control of AR (6, 16).

Finally, the role of AIT in the primary PAT is currently under investigation. In a recent proof of concept study, 111 young children (aged 5–9 months), not sensitized, but at high risk of atopy, were treated with prophylactic HDM SLIT (80). A significant sensitization prevention was demonstrated in the active group; however, no significant preventive effect was observed on HDM sensitization or allergy-related symptoms (80). Further studies are expected to clarify the role of AIT as early-intervention in high-risk children.

## Patient selection and biomarkers of response

Since AIT is allergen-specific, a detailed clinical history and appropriate allergy diagnostic tests are essential to properly identify the triggering clinical relevant allergen(s) (2). In case of polysensitized patients, the identification of major allergens should be supported by the use of component-resolved diagnostics (81). Uncontrolled AR symptoms despite antihistamines and/or topical corticosteroids and allergen avoidance measures and/or side-effects of medication, the duration of AR symptoms, as well as the assessment of asthma control, are essential to consider AIT as a therapeutic option (2). Each patient should be evaluated individually by considering the benefits and the risks, and the ability to comply/cooperate with AIT (26). Although some clinical studies have demonstrated efficacy and safety of AIT in preschool children (82, 83), there is no consensus on a specific lower age limit for initiating AIT for respiratory allergy (8). However, this issue still deserves further extensive studies in the perspective of preventive strategies.

Although highly effective, some patients could not respond to AIT treatment. Thus, the identification and validation of potential predictive biomarkers of AIT effectiveness is an active filed of research and could enhance the selection and the clinical management of patients receiving AIT. Biomarkers are quantitative measurement predicting clinical and immunological effects of AIT (84). In particular, ideal biomarkers for AIT should assist in patient selection and identification of responders and predict clinical and immunological response during treatment and after discontinuation of treatment (84). The EAACI Taskforce recently reviewed all candidate biomarkers used in clinical trials of AR patients with or without asthma (18, 84) (Table 2). Markers can be cellular (Tregs), humoral (allergen-specific IgG_4_ (sIgG_4_), IgE/IgG_4_), molecular (interleukins), or functional (IgE FAB and blocking factor). Although several studies have included biomarkers as secondary outcomes, specifically AIT biomarker studies are still lacking. To date, raised serum allergen-specific IgE (sIgE) are considered the only useful biomarker to select candidates for AIT, in the context of a clear history of symptoms on exposure to the relevant allergen (84). In particular, preliminary data suggest that higher levels of sIgE in children could be helpful to predict AIT efficacy (85). Furthermore, sIgG4, sIgE/total IgE ratio, and IgE-FAB are in the pipeline as candidate biomarkers for compliance and response to AIT, respectively (84). More research is needed to confirm and interpret the possible association of biomarkers with both clinical response and persistence of clinical benefit after discontinuation of AIT.

**Table 2:** Potential biomarkers for allergen immunotherapy (AIT).

**Categories**	**Candidate biomarkers**	**Domain**	**Advantages**	**Disadvantages, unmet need**	**Possible applications**
Biomarkers for diagnosis	IgE (sIgE, tIgE, sIgE/tIgE)	Antibodies	Elevated serum IgE levels in the context of a clear history of allergic symptoms is a biomarker for selection of patients for AIT	No clear correlation with clinical outcome Lack of validation in RDBPCT Lack of standardization of assay platform and reference ranges/cut-off values	Prediction of disease severity and/or progression
	CD63, CD203c, DAO, basophil histamine release				
Biomarkers predictive of AIT safety	CD63, CD203c, DAO, basophil histamine release	Basophil activation	Small amount of blood (<2 ml) is required to perform the test	Mechanism of allergen induced basophil hyperresponsiveness during AIT not completely known Limited number of studies Need for standardized assays	Reduced risk of side effects and improvement of patients' compliance to treatment
Biomarkers of AIT efficacy	IgG subclasses (sIgG_1_, sIgG_4_, sIgE/IgG_4_)	Antibodies	sIgG_4_ is a biomarker of immunologic response of AIT	No clear correlation with clinical outcome Limited data on local antibody levels and activities	Prediction of patients' compliance
	sIgE/total IgE	Antibodies	Potential positive predictive biomarker of response for AIT	Lack of validation in RDBPC	Prediction of clinical response
	IgE FAB, IgE-BF	Serum Inhibitory activity for IgE	Highly reproducible serum-based assay Association with clinical outcome has been reported in some studies	Availability limited to specialized centers or laboratories	
Not yet determined	CCR3, ECP, eotaxin, IFN-γ, IL-2, IL-2R, IL-4/5/6, IL-8/9/10, IL-13/18, MCP-1, TARC, transthyretin	Cytokines and chemokines	May be useful to further explore mechanisms of AIT	No correlation with clinical outcome	Not known
	DCs, Breg, Treg	Cellular biomarkers	Early biomarkers of immunologic response	Not routinely performed, limited applications in clinical practice No clear correlation with clinical outcome	Prediction of immunological response
	SPT, Id, In, chamber studies	*In vivo* biomarkers	Provocation tests used as surrogate biomarkers of clinical response to AIT Provocation methods are recommended as primary endpoints in proof-of-concept and dose-finding trials of AIT	Standardization and validation differ from the various challenge protocols Comparison between provocation test results and symptoms after natural exposure are currently lacking	Prediction of clinical response

*Adapted from Shamji et al. (84). BF, binding factor; Breg, B regulatory cell; CCR, chemokine receptor; CD, cluster of differentiation; DAO, diamine oxidase; DC, dendritic cell; ECP, eosinophil cationic protein; FAB, facilitated antigen binding; Id, intradermal test; IFN, interferon; Ig, immunoglobulin; IL, interleukin; In, intranasal test; MCP, monocyte chemoattractive protein; RDBPCT, Randomized double-blinded placebo-controlled trial; sIg, allergen-specific Ig; SPT, skin prick test; TARC, thymus and activation-regulated chemokine; tIg, total Ig; Treg, T regulatory cell*.

## Conclusions

AIT represents a valuable therapeutic option, especially in childhood, to modify the progression of allergic disease. AIT may be particularly useful in children with AR and new-onset asthma because it may modify the long-term prognosis of their airway disease. Both SCIT and SLIT seem to be effective in pediatric allergic asthma, showing a promising steroid-sparing effect of which patients treated with high-dose pharmacotherapy for a long term could benefit most. To date, uncontrolled asthma remains a clear contraindication for AIT treatment; however, coupling novel biological therapies with AIT could represent a novel approach to treat these patients with high risk of adverse reactions. Over the last decade considerable advances in the AIT approach have been also made to move forward this therapeutic field in the context of personalized medicine. The advanced knowledge of the mechanisms of sustaining clinical and immunological tolerance toward allergens, the implementation of vaccination strategies (using recombinant allergen extracts, or modified extracts at increased safety/efficacy, or adjuvants to further stimulate the immune system), the implementation and diffusion of international guidelines, the definition of regulatory aspects such as standardization and registration of AIT products, the standardization of clinical trial outcomes, as well as the planning of future dedicated pediatric studies, will all implement the evidence of efficacy and safety of AIT for allergic children.

## Author contributions

All authors made substantial contribution to the conception of the work, reviewed the literature on the subject, and drafted the final version of the manuscript. AL and MT revised it critically for important intellectual content. All authors finally approved the version to be published and agreed to be accountable for all aspects of the work in ensuring that questions related to the accuracy or integrity of any part of the work are appropriately investigated and resolved.

### Conflict of interest statement

The authors declare that the research was conducted in the absence of any commercial or financial relationships that could be construed as a potential conflict of interest.
